# LncRNA RP11-551L14.4 suppresses breast cancer development by inhibiting the expression of miR-4472

**DOI:** 10.7717/peerj.14482

**Published:** 2022-12-06

**Authors:** Bin Wang, Hang Chen, Rui Yang, Lei Xing, Chuan Chen, Junxia Chen

**Affiliations:** 1 Department of Cell Biology and Genetics, Chongqing Medical University, Chongqing, China; 2 Department of Oncology, Daping Hospital, Army Medical University, Chongqing, China; 3 Department of Endocrine and Breast Surgery, The First Affiliated Hospital of Chongqing Medical University, Chongqing, China

**Keywords:** RP11-551L14.4, miR-4472, Breast cancer, Proliferation

## Abstract

**Background:**

Previous studies have been reported that long non-coding RNA (lncRNA) can regulate the expression of genes which are involved in many important cellular processes The potential role of lncRNA RP11-551L14.4 in the development of breast cancer and the possible regulatory mechanisms was investigated.

**Methods:**

Quantitative real-time polymerase chain reaction (qRT-PCR) was conducted to analyze RP11-551L14.4 expression in 36 paired breast cancer tissues and adjacent tissues. The expression of RP11-551L14.4 in multiple breast cancer cell lines was detected by qRT-PCR. Meanwhile, overexpression of RP11-551L14.4 models was established using lentivirus in BT474 and T47D breast cancer cells. Cell counting kit-8 (CCK-8), cell colony formation and cell cycle assays were performed to detect the effects of RP11-551L14.4 on the biological function of breast cancer cells. Besides, bioinformatics techniques, dual luciferase reporter gene assay and rescue experiments were used to investigate the potential mechanisms.

**Results:**

RP11-551L14.4 expression was negatively associated with the advanced tumor stage. Breast cancer patients with low RP11-551L14.4 expression manifested a poorer prognosis. The results of qRT-PCR showed that RP11-551L14.4 expression in breast cancer tissues was significantly lower than in adjacent tissues. Meanwhile, overexpression of RP11-551L14.4 significantly decreased the cell proliferation and cell cycle. Bioinformatics technology showed that RP11-551L14.4 could complementarily bind to miR-4472. qRT-PCR results indicated that the expression levels of miR-4472 and RP11-551L14.4 in breast cancer were negatively correlated. Luciferase reporter gene assay showed that miR-4472 remarkably decreased the relative luciferase activity of the wild-type RP11-551L14.4 vector. miR-4472 is a direct target gene of RP11-551L14.4. miR-4472 levels were reduced, and repulsive guidance molecule A (RGMA) mRNA or protein levels were increased after overexpression of RP11-551L14.4 in the breast cancer cells. miR-4472 reversed the effects caused by RP11-551L14.4 in breast cancer cells.

**Conclusion:**

RP11-551L14.4 expression was remarkably decreased in breast cancer tissues and cells. RP11-551L14.4 may inhibit the malignant progression of breast cancer by regulating miR-4472 expression.

## Introduction

Breast cancer is derived from breast epithelial cells and ranks as a common malignant cancer around the world (Lakshmi, Hughes & Priya, 2021; Nagini, 2017; Shi et al., 2020a). A large portion of patients are in advanced stage by the time of diagnosis due to its rapid progression (Li, Jin & Li, 2021a). The long-term prognosis of breast cancer patients is poor, which makes breast cancer a threat to human health (Li et al., 2020a). The process of breast cancer occurrence involves genetic and epigenetic changes (Dong et al., 2019; Shi et al., 2020b; Yin et al., 2020). Hence, it is urgent to clarify the potential mechanisms of tumorigenesis, so as to search for new molecular biomarkers for early diagnosis and prognosis.

Long non-coding RNAs (lncRNAs) are single-stranded and non-coding RNA molecules (more than 200 nucleotides) (Chen et al., 2020). lncRNAs are localized in the nucleus or cytoplasm, which are transcribed by RNA polymerase II (Han et al., 2020). lncRNAs can regulate the expression of genes which are involved in many important cellular processes such as cell proliferation, invasion, migration, apoptosis and autophagy (Kong et al., 2019; Yousefi et al., 2020; Zhao et al., 2018). lncRNAs could act as tumor suppressor or novel oncogenes in cancers (Li, Zhang & Li, 2020c). It has been demonstrated that some lncRNAs are related to the occurrence and progression of breast cancer (Liang et al., 2020; Xiu et al., 2019; Zhang et al., 2020b). According to the Ensembl database (Hunt et al., 2018), RP11-551L14.4 is an lncRNA with a length of 623 nt. The expression of RP11-551L14.4 and its role in the progression of cancer has not been reported. The Lnc2Cancer 3.0 database showed that RP11-551L14.4 was downregulated in breast cancer tissues compared to normal breast tissues.

In the current study, we demonstrated that RP11-551L14.4 was significantly low-expressed in breast cancer tissues and cell line models. Patients with high RP11-551L14.4 expression displayed favorable overall survival and disease-free survival. Notably, *in vitro* experiments revealed that overexpression of RP11-551L14.4 inhibited breast cancer cell growth. In addition, we also identified miR-4472 as a target of RP11-551L14.4. RP11-551L14.4 acts as a tumor suppressor in breast cancer and may be a new molecular marker for diagnosis and therapeutic evaluation of breast cancer in the future.

## Material and Methods

### Tissue specimens

In this study, 36 pairs of breast cancer tissues (ICD-10 code: 2C60) and corresponding adjacent non-tumor tissues were collected from surgically treated breast cancer cases (median age, 56 years old; age range, 45–73 years old) at the Department of Endocrine and Breast Surgery, The First Affiliated Hospital of Chongqing Medical University between January 2020 and August 2021. All tissues are stored in liquid nitrogen. Patients with other diseases were excluded and only patients with a single breast cancer were retained. The collection of tissue specimens was approved by the Ethics Committee of Chongqing Medical University (Approval No. 20200613). All patients have signed informed consent.

### Cell culture

The human breast cancer cell lines (MDA-MB-453, MCF7, BT474, T47D, ZR-75-1) and the normal human breast epithelial cell line (MCF-10A) were purchased from the American Type Culture Collection (ATCC, Manassas, VA, USA). ZR-75-1, T47D and BT474 were cultured in Roswell Park Memorial Institute-1640 (RPMI-1640, Gibco; Thermo Fisher Scientific, Waltham, MA, USA) containing 10% fetal bovine serum (FBS, Gibco; Thermo Fisher Scientific, Waltham, MA, USA); MDA-MB-453, MCF7 and MCF-10A were cultured in Dulbecco’s Modified Eagle’s medium (DMEM, Gibco; Thermo Fisher Scientific) containing 10% FBS. All of them were maintained in an incubator at 37 °C with 5% CO_2_.

### Transfection assay

The lentivirus containing RP11-551L14.4 overexpression sequences (RP11-551L14.4) and the negative control (NC) were purchased from GenePharma (Shanghai, China). Cells were seeded into 6-well plates and infected with lentivirus. After 48 h, cells were harvested and subjected to Reverse transcription-quantitative polymerase chain reaction (qRT-PCR), Western blotting and cell function assays.

*Bioinformatics analysis*. The expression profile for RP11-551L14.4 was obtained from Lnc2Cancer 3.0 database (http://bio-bigdata.hrbmu.edu.cn/lnc2cancer/) (Gao et al., 2021). The survival analysis for RP11-551L14.4 was obtained from the Gene Expression Profiling Interactive Analysis (GEPIA) database (http://gepia.cancer-pku.cn/index.html) (Tang et al., 2017). The LncBase Predicted v.2 database (http://diana.imis.athena-innovation.gr) (Paraskevopoulou & Hatzigeorgiou, 2016) was used to predict the target miRNA of RP11-551L14.4.

### qRT-PCR

Total RNA was isolated from tissues and cells using TRIzol reagent (Invitrogen, Carlsbad, CA, USA) and then reverse transcribed into complementary Deoxy-ribose Nucleic Acid (cDNA) using PrimeScript RT Master Mix (TaKaRa, Otsu, Shiga, Japan). qRT-PCR was conducted using SYBR Premix Ex TaqTM (TaKaRa, Otsu, Shiga, Japan) according to the manufacturer’s protocol. The primers are listed below: RP11-551L14.4: F: 5′-CTTGGCCTTCTCCATAACCA-3′, R: 5′-GACTGCTATGGAGGCTGAGG-3′; Glyceraldehyde 3-phosphate dehydrogenase (GAPDH): F: 5′-AAGGTGAAGGTCGGAGTCAAC-3′, R: 5′-GGGGTCATTGATGGCAACAATA-3′; *RGMA*: F: 5′- AACCAGCAGATCGACTTCCAG-3′, R: 5′- ACGGCTGTCTCGTATGGGA -3′; U6: F: 5′-CTCGCTTCGGCAGCACA-3′, R: 5′-AACGCTTCACGAATTTGCGT-3′; miR-4472: F: 5′-AAAACAACACCCCCCACC-3′, R: 5′-CTCAACTGGTGTCGTGGA -3′; GAPDH was used for normalization for RP11-551L14.4 and *RGMA* mRNA. U6 was used for normalization for miR-4472.

### Cell proliferation assay

After infection, T47D and BT474 cells were seeded into 96-well plates at 3,000 cells per well and maintained over 120 h duration, 20 µL cell counting kit-8 (CCK-8) (Dojindo Laboratories, Kumamoto, Japan) was added to each well and incubated for 2 h. Optical density (OD) value was measured at a wavelength of 450 nm by the spectrophotometer.

### Colony formation assay

The infected T47D and BT474 cells were collected and seeded at 1,000 cells into 6-well plates and maintained for 14 days. The cells were washed three times with phosphate-buffered saline (PBS; Thermo Fisher Scientific), then fixed in methanol for 30 min and stained with with crystal violet staining solution (Sigma-Aldrich, St. Louis, MO, USA) for 30 min. Colonies were photographed and counted.

### Cell cycle assay

The infected T47D and BT474 cells were harvested, washed with (PBS; Thermo Fisher Scientific) and then stained with propidium iodide (PI) solution for 30 min. Flow cytometry analyses were performed by using a flow cytometer (FACScan^^®^^; BD Biosciences) and the Modfit LT software (Verity Software House, US).

### Luciferase reporter assay

The binding sites of RP11-551L14.4 and miR-4472 were predicted with the LncBase Predicted v.2 database. The RP11-551L14.4-wild-type (RP11-551L14.4-WT) luciferase reporter gene plasmid and the RP11-551L14.4-mutant (RP11-551L14.4-MUT) reporter gene plasmid were constructed by GenePharma (Shanghai, China). T47D cells were co-transfected with miR-4472 mimic or miR-NC, and with RP11-551L14.4-WT or RP11-551L14.4-MUT reporter gene plasmid using Lipofectamine 3000 reagent (Invitrogen). After incubated for 48 h, the intensity of the fluorescence was measured using dual-luciferase activity assay according to the manufacturer protocol (Promega, Madison, WI, USA). The following sequences were used: miR-4472 mimic, 5′-GGUGGGGGGUGUUGUUUU-3′; miR-NC mimic, 5 ′-UUGUCCGAACGUGUCACGU-3′.

### Western blotting

Total proteins were isolated with RIPA buffer (Thermo Fisher Scientific). After separated by 10% SDS-PAGE, proteins were transferred onto polyvinylidene difluoride membranes (Millipore, Billerica, MA, USA). Then membranes were blocked with 5% BSA for 2 h at room temperature and incubated with primary antibodies at 4 °C for 12 h. The antibodies are as follows: RGMA (cat. no. ab169761), CDK4 (cat. no. ab108357), CDK6 (cat. no. ab124821), Cyclin D1 (cat. no. ab16663), Cyclin D2 (cat. no. ab207604), GAPDH (cat. no. ab9485) (all 1:2,000 dilution; Abcam, Cambridge, UK). After washing with TBST-Tween-20), the membranes were incubated with secondary antibodies for 2 h. The antibodies are as follows: HRP-conjugated goat anti-rabbit IgG (cat. no. ab150077) (both 1:10,000 dilution; Abcam, Inc.). Protein bands were visualized using ECL (GE Healthcare, Chicago, IL, USA). GAPDH was used as a loading control.

### Statistical analysis

SPSS software (version 20.0; IBM Corp.) was used to perform the statistical analysis. Data are shown as the mean ± standard deviation. All comparisons between paired tumor and adjacent non-tumor tissues were performed with paired Student’s t-tests. Unpaired Student’s t-tests were performed for comparisons between two groups of cells. Cox proportional hazards model multivariate analyses were used to evaluate the significance of lncRNA RP11-551L14.4 expression and clinicopathological features. The overall survival or disease free survival was assessed using Kaplan–Meier survival analysis followed by the log-rank test. One-way ANOVA followed by Bonferroni’s correction was adopted to compare several groups. The gene expression correlation was assessed using the Pearson’s correlation coefficient test. *P* value<0.05 was considered statistically significant.

## Results

### RP11-551L14.4 is low expressed in breast cancer tissues and cell lines

**Figure 1:**
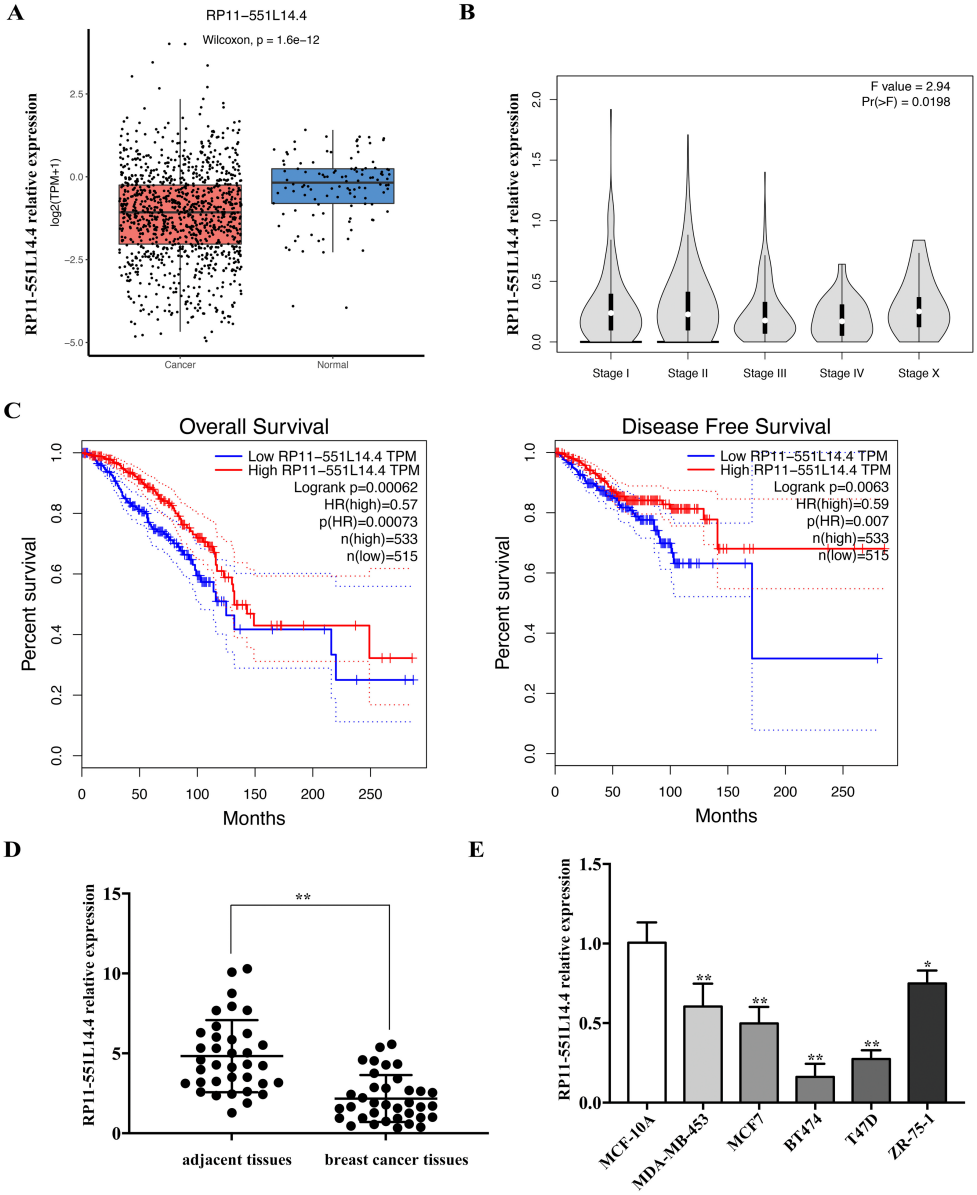
(A–E) RP11-551L14.4 is lowly expressed in breast cancer.

We used the Lnc2Cancer 3.0 database to analyze the expression of RP11-551L14.4 in breast cancer and found that RP11-551L14.4 were low expressed in breast cancer tissues compared to normal breast tissues (Fig. 1A) and the expression of RP11-551L14.4 decreased with advanced tumor stage (Fig. 1B). The Kaplan–Meier survival curves indicated that patients with high RP11-551L14.4 expression displayed favorable overall survival and disease-free survival (Fig. 1C). To verify the expression of RP11-551L14.4 in breast cancer, we collected 36 pairs of breast cancer tissues and adjacent tissues. The results of qRT-PCR showed that the expression of RP11-551L14.4 decreased in breast cancer tissues when compared to the adjacent tissues (Fig. 1D). Consistently, RP11-551L14.4 was found low expressed in breast cancer cell lines (MDA-MB-453, MCF7, BT474, T47D, ZR-75-1). Among them, RP11-551L14.4 has the lowest expression in T47D and BT474; therefore, the two cell lines were selected for subsequent experiments (Fig. 1E). Collectively, the results suggested that RP11-551L14.4 might act as a tumor suppressor in breast cancer.

### RP11-551L14.4 overexpression inhibits breast cancer cells proliferation, colony formation and attenuates cell cycle

**Figure 2:**
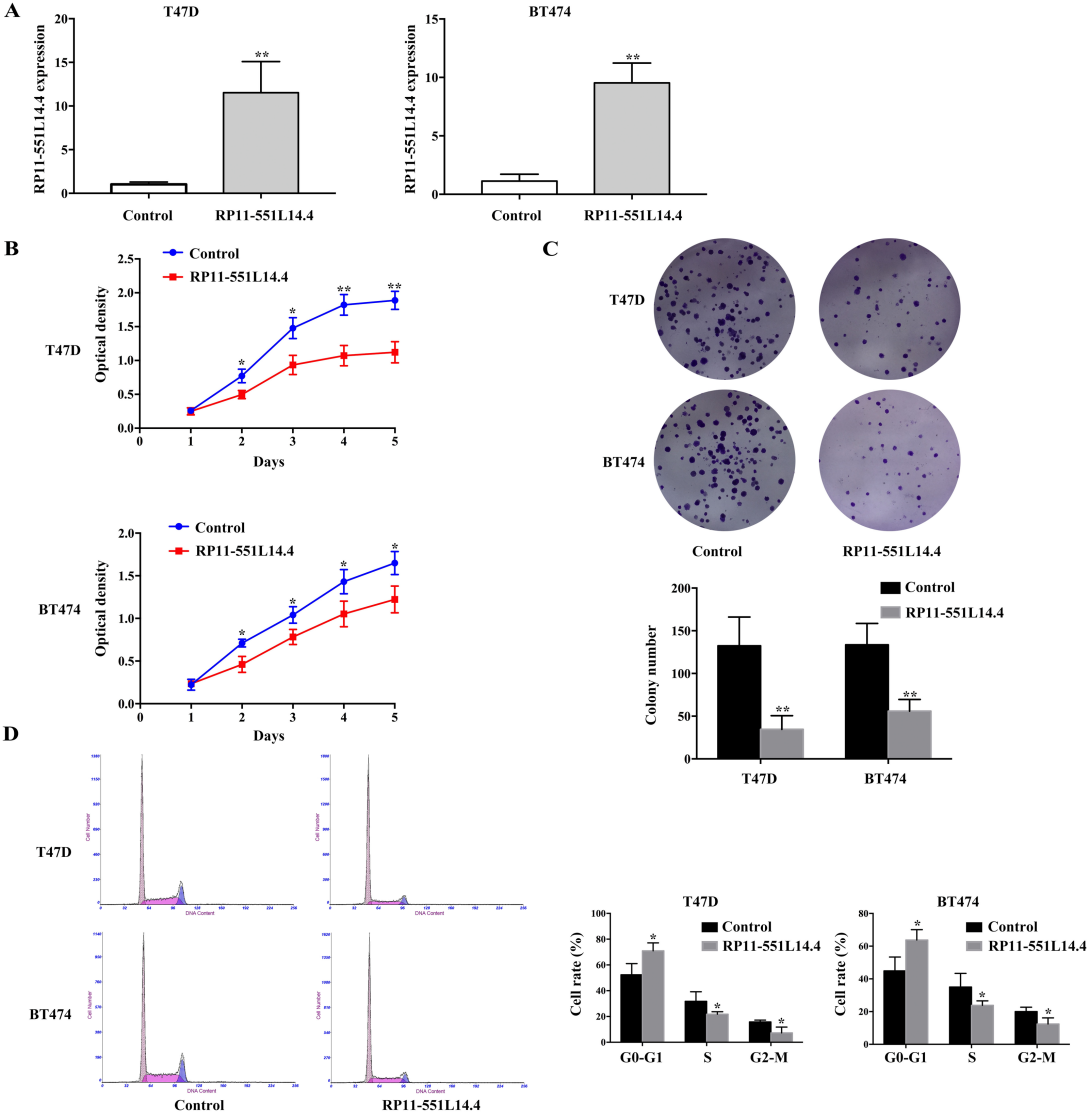
(A–D) RRP11-551L14.4 overexpression inhibits breast cancer cells proliferation, colony formation and cell cycle.

To investigate the function of RP11-551L14.4 in breast cancer, we constructed RP11-551L14.4 overexpression model using lentiviral vector. qRT-PCR was performed to detect the level of RP11-551L14.4 after transfecting the RP11-551L14.4 lentiviral vector in T47D and BT474 (Fig. 2A). Cell proliferation and clonogenic assays were analyzed by CCK-8 and cell cloning assays in RP11-551L14.4 overexpressing cell lines, respectively. The proliferation and colony-forming abilities of T47D and BT474 cells were significantly inhibited (Figs. 2B and 2C). Flow cytometry assay showed that the G_0_/G_1_ phase fraction of the RP11-551L14.4 group was more than that of the Control group, but less cells in the G_2_/M phase (Fig. 2D). The results revealed that cell proliferation and cell cycle in RP11-551L14.4 overexpression group was remarkably suppressed compared with that in the Control group.

### miR-4472 is a direct target of RP11-551L14.4

**Figure 3:**
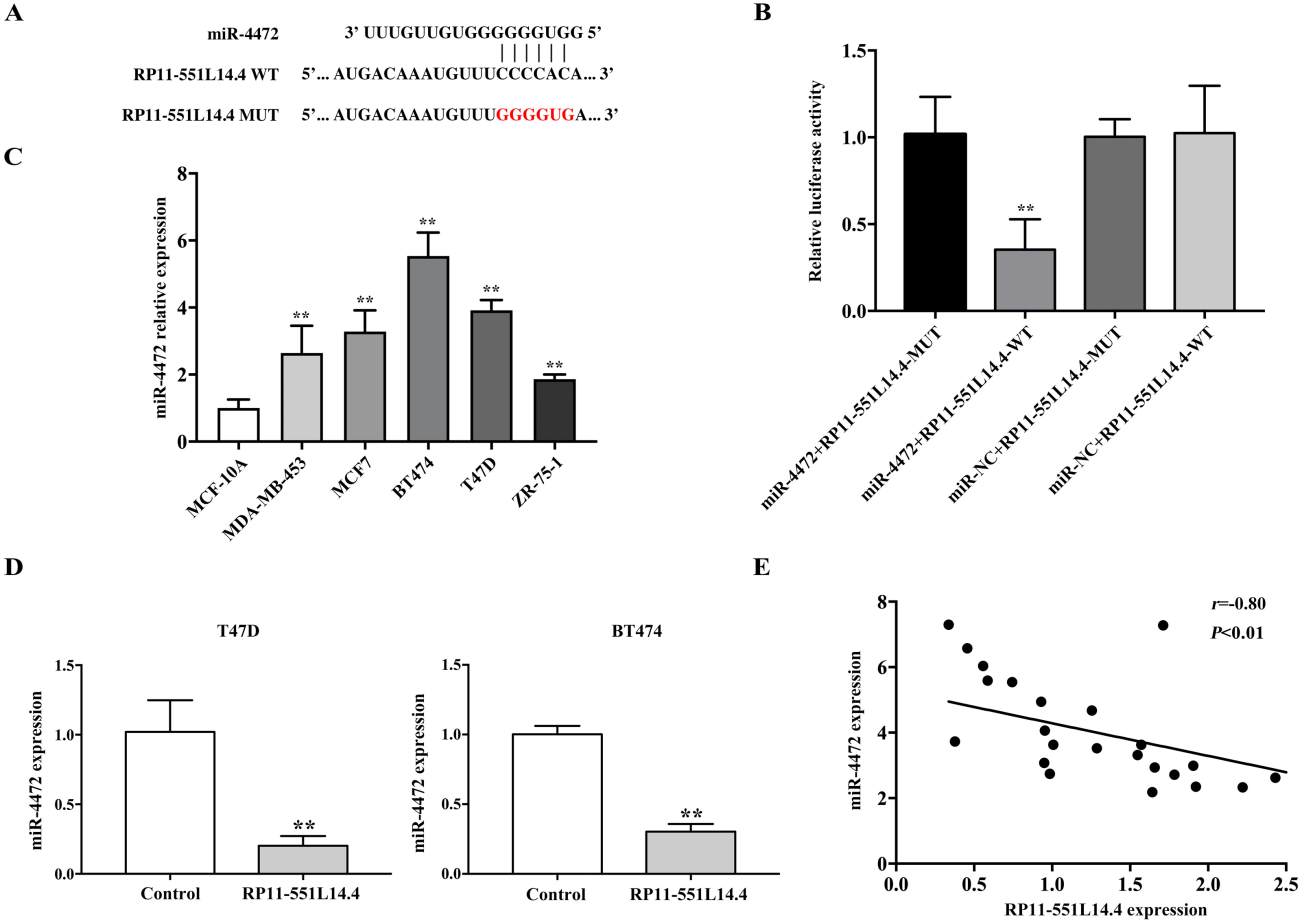
(A–E) MiR-4472 is a direct target of RP11-551L14.4.

The LncBase Predicted v.2 database was used to investigate the underlying mechanisms by which RP11-551L14.4 exerts its function. miR-4472 was predicted as a potential target of RP11-551L14.4 (Fig. 3A). Dual luciferase reporter assay revealed that miR-4472 significantly impaired the luciferase activity of the wild type RP11-551L14.4 vector without impairing that of the mutant vector (Fig. 3B). In addition, miR-4472 was significantly upregulated in breast cancer cell lines (Fig. 3C). qRT-PCR showed that miR-4472 expression was attenuated after transfecting the RP11-551L14.4 lentiviral vector (Fig. 3D). Correlation analysis between RP11-551L14.4 and miR-4472 expression in breast cancer tissues revealed an inverse relationship (Fig. 3E). The above results suggested that miR-4472 can be targeted by RP11-551L14.4 through the binding site.

### miR-4472 reverses the effects caused by RP11-551L14.4 in breast cancer cells

**Figure 4:**
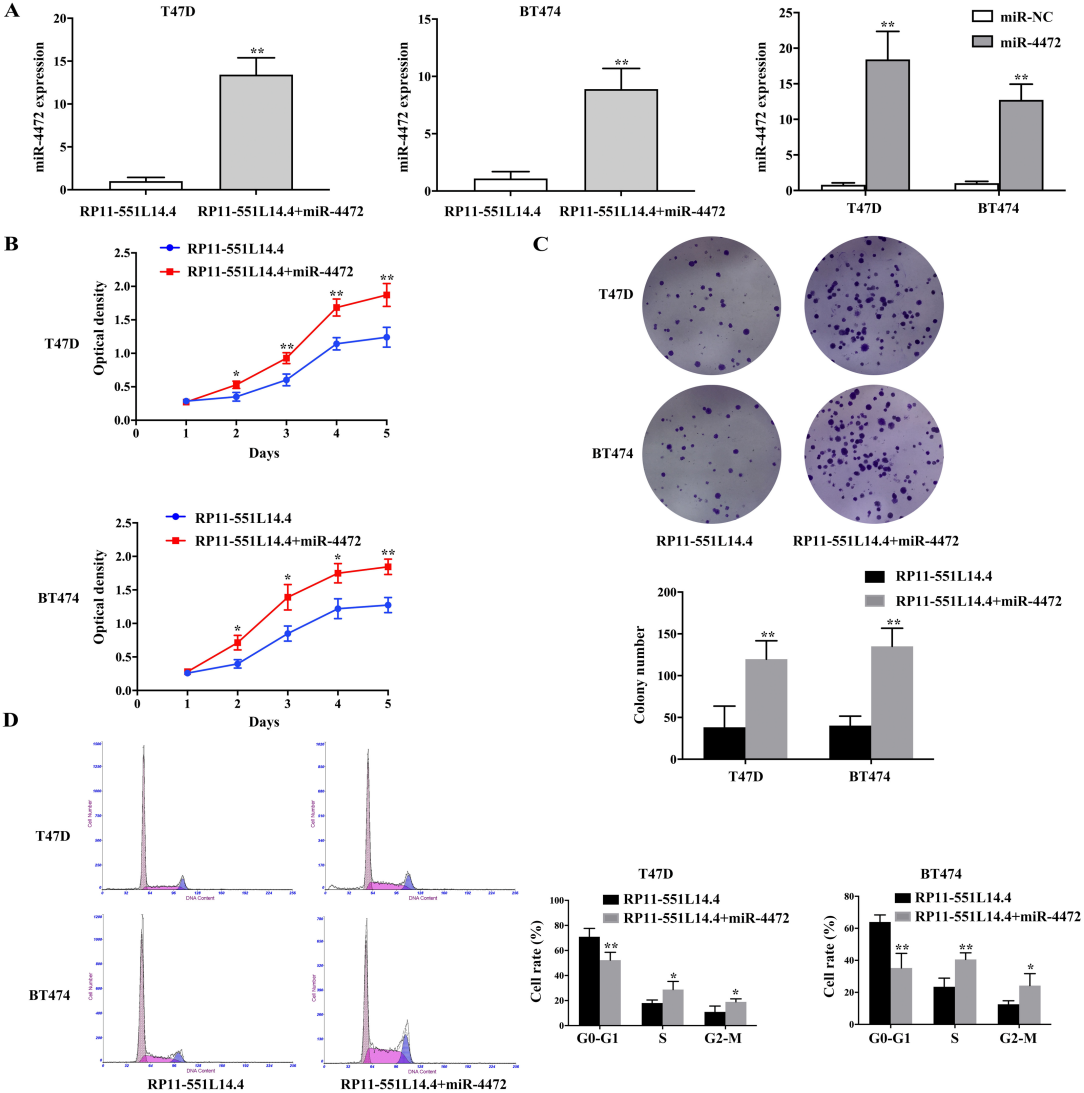
(A–D) MiR-4472 neutralized the anti-cancer effect of RP11-551L14.4.

Considering that RP11-551L14.4 could negatively regulate the expression of miR-4472, we supposed that RP11-551L14.4-mediated downregulation of miR-4472 directly decreased breast cancer cell proliferation. A rescue experiment was conducted, both RP11-551L14.4-overexpressed vector and miR-4472 mimics were co-transfected into breast cancer cells. qRT-PCR was performed to detect the level of miR-4472 after transfecting miR-4472 mimics with or without RP11-551L14.4-overexpressed vector in T47D and BT474 (Fig. 4A). The rescue experiments showed that restoration of miR-4472 could abrogate RP11-551L14.4-induced suppression of proliferation in both T47D and BT474 cells (Figs. 4B–4D). Therefore, our results support the concept that RP11-551L14.4 function as a tumor gene suppressor since its effect could be reversed by miR-4472 in breast cancer cells.

### RP11-551L14.4 promotes repulsive guidance molecule A (RGMA) expression

**Figure 5:**
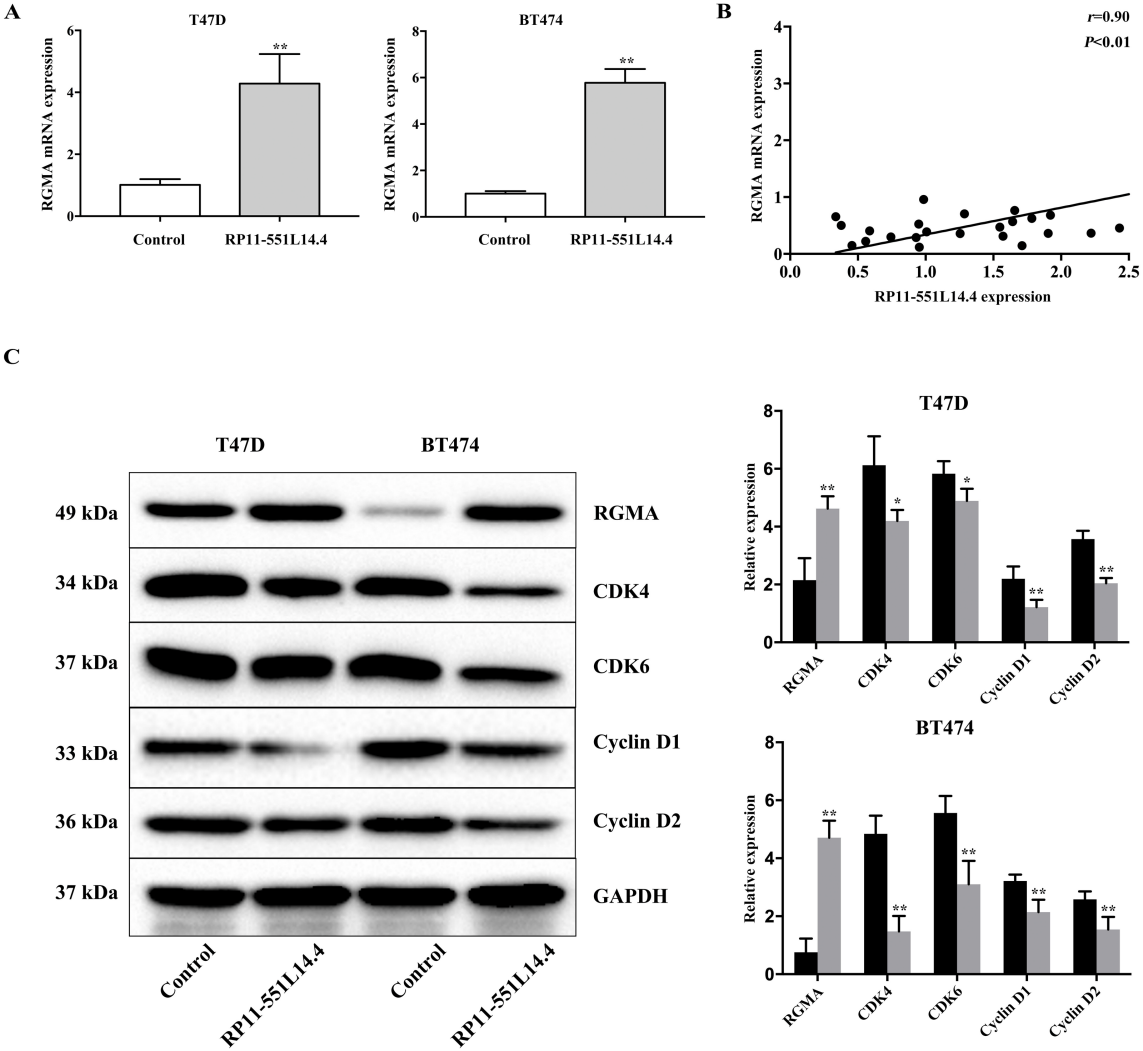
(A–C) Impact of RP11-551L14.4 on *RGMA* and cell cycle related proteins expression.

qRT-PCR and Western blot showed that RGMA expression was enhanced after transfecting the RP11-551L14.4 lentiviral vector (Figs. 5A and 5C). Correlation analysis between RP11-551L14.4 and *RGMA* mRNA expression in breast cancer tissues revealed a positive relationship (Fig. 5B). Western blot analysis showed that the expression of cell cycle related proteins CDK4, CDK6, Cyclin D1 and Cyclin D2 was attenuated in the RP11-551L14.4 group compared with the Control group (Fig. 5C).

## Discussion

lncRNAs are a class of non-coding RNAs which can regulate gene expression in many ways, such as binding to a protein-binding partner and regulate the localization of proteins in subcellular cells (Liu et al., 2020; Zhang et al., 2020c; Zhang et al., 2020d). Besides, lncRNAs can affect the transcription process of regulatory proteins (Lu et al., 2018). lncRNAs can regulate a variety of biological functions including gene imprinting, cell cycle regulation, translational (Ma et al., 2020). It is well acknowledged that abnormally expressed lncRNAs can act on the occurrence and progression of cancers by affecting many aspects of epigenetics (Qin et al., 2019; Sharma et al., 2021; Shen, Peng & Shen, 2020). The expression of RP11-551L14.4 and its specific mechanism in breast cancer still remains elusive. The Lnc2Cancer 3.0 database showed that RP11-551L14.4 was downregulated in breast cancer tissues and negatively correlated with breast cancer grades. In addition, patients with higher RP11-551L14.4 expression have better overall survival and disease-free survival. We speculate that RP11-551L14.4 may suppress cancer progression in breast cancer. In this study, we revealed that RP11-551L14.4 is decreased in breast cancer tissues. Similarly, such a decrease was also found in several cell models established from breast cancer biopsies. CCK-8 and cell cloning experiments confirmed that the up-regulation of RP11-551L14.4 can indeed attenuate the malignant progression of breast cancer. Meanwhile, the decreased expression of cell cycle-related proteins in breast cancer cells suggested that RP11-551L14.4 could inhibit the growth of breast cancer cells.

lncRNAs have been demonstrated to fuction as ‘miRNA sponges’ and compete with miRNA for binding to target genes of miRNA, thus up-regulate the expression of miRNA target genes (Wu et al., 2021; Zhang et al., 2020a; Zhang, Guan & Tang, 2021; Zhou et al., 2021). We further adopted bioinformatics to analyze miRNAs that RP11-551L14.4 can bind with. The LncBase Predicted v.2 database showed that RP11-551L14.4 had putative binding sites with miR-4472. miR-4472 has been verified to act as an oncogene in different kinds of cancers (Li et al., 2020b). Li et al. (2020b) found that miR-4472 was significantly highly expressed in breast cancer tissues and cell lines, the enrichment of miR-4472 in breast cancer cells could promote tumor growth *in vivo* and knockdown of miR-4472 suppressed cell proliferation. Luciferase activity assay confirmed direct targeting of miR-4472 by RP11-551L14.4. Additionally, correlation analysis between RP11-551L14.4 and miR-4472 expression in breast cancer tissues revealed an inverse relationship. Overall, the present study indicated that RP11-551L14.4 can specifically target miR-4472, and down-regulate miR-4472 expression. As reported, miR-4472 contributes to breast cancer cell growth by inhibiting the expression of *RGMA* gene (Li et al., 2020b). The RGMA protein, a glycosylphosphatidylinositol-anchored cell membrane-associated protein, functions as a tumor suppressor in a variety of cancers including breast cancer, colon cancer and gallbladder cancer (Li et al., 2021b; Lu et al., 2019; Song et al., 2021). As been reported, RGMA protein can attenuate cellular proliferation and cell cycle of breast cancer cells (Li et al., 2021b). In our study, we revealed that RP11-551L14.4 overexpression in breast cancer cells leads to an increase of *RGMA* expression. Notably, correlation analysis between RP11-551L14.4 and *RGMA* mRNA expression in breast cancer tissues revealed a positive relationship. These results suggested that further demonstrated that RP11-551L14.4 functions by regulating miR-4472 expression. There were several limitations in the study. The effect of RP11-551L14.4 on breast cancer cell proliferation *in vivo* should be explored. It is unknown whether RP11-551L14.4 affects the radiosensitivity of breast cancer cells. Moreover, the role of RP11-551L14.4 in breast cancer cell metastasis should be investigated.

In summary, we showed that RP11-551L14.4 is significantly decreased in breast cancer tissues and cells. Breast cancer patients with high RP11-551L14.4 expression displayed favorable overall survival and disease-free survival. Moreover, RP11-551L14.4 may inhibit the proliferation of breast cancer cell growth by modulating miR-4472 expression. RP11-551L14.4 could be considered as a tumour suppressor in breast cancer cells and may provide a potentially significant therapeutic target for breast cancer.

## Abbreviations

lncRNAs: long non-coding RNAs
miR-4472: microRNA-4472
MUT: mutant type
WT: wild type
*RGMA*
: repulsive guidance molecule A

##  Supplemental Information

10.7717/peerj.14482/supp-1 Supplemental Information 1 Original western blots

10.7717/peerj.14482/supp-2 Supplemental Information 2 Raw data

## Additional Information and Declarations

### Data Availability

The following information was supplied regarding data availability: The raw data and uncropped blots are available in the Supplemental Files.
