# Supplementary figures and images for: LncRNA RP11-551L14.4 suppresses breast cancer development by inhibiting the expression of miR-4472
Source: PeerJ. 2022 Dec 6;10:e14482. doi: 10.7717/peerj.14482 (PMC9745927; doi:10.7717/peerj.14482)

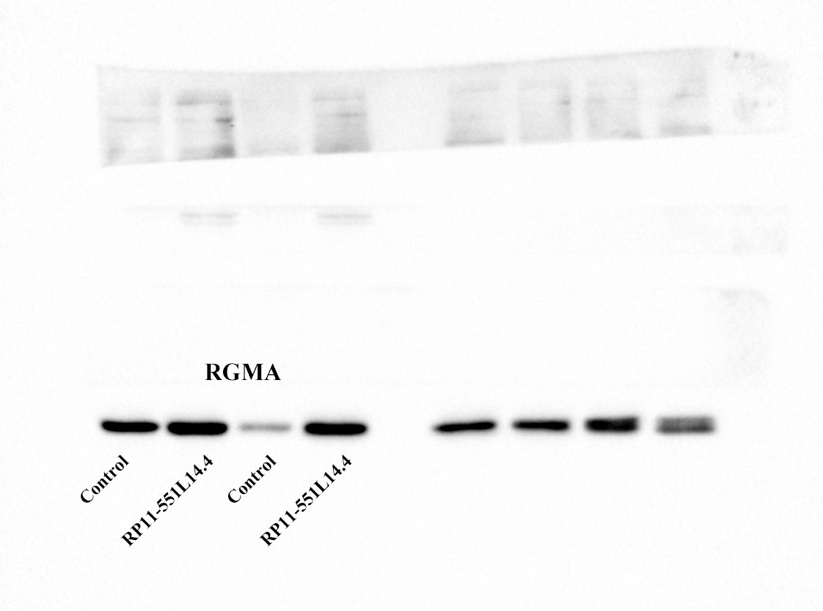


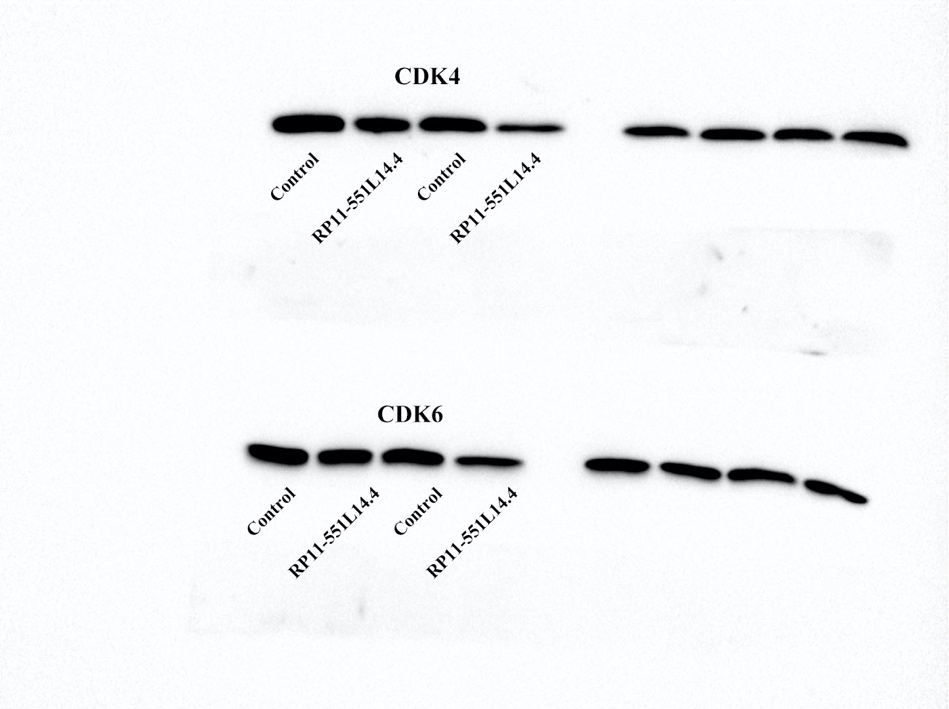


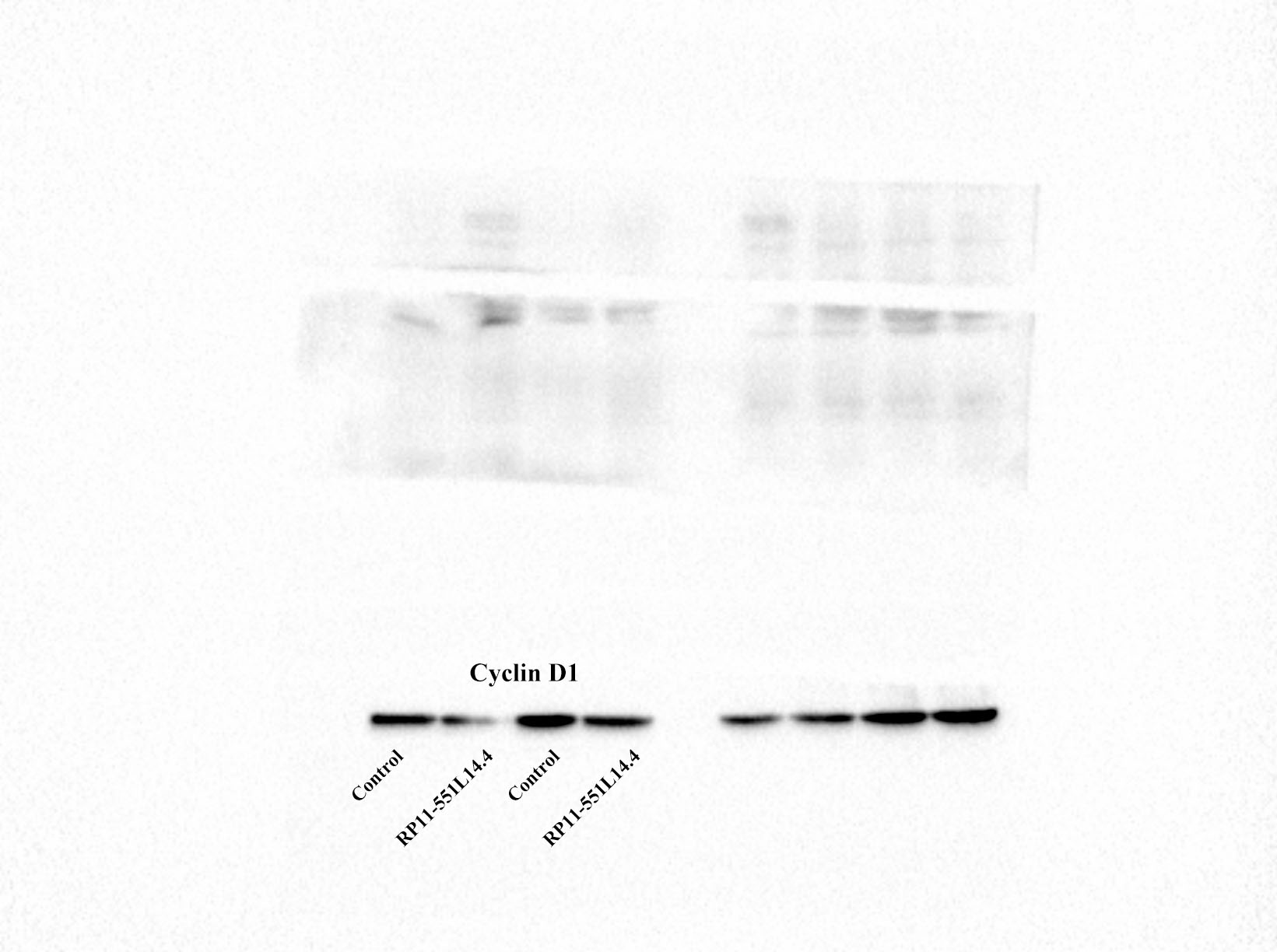


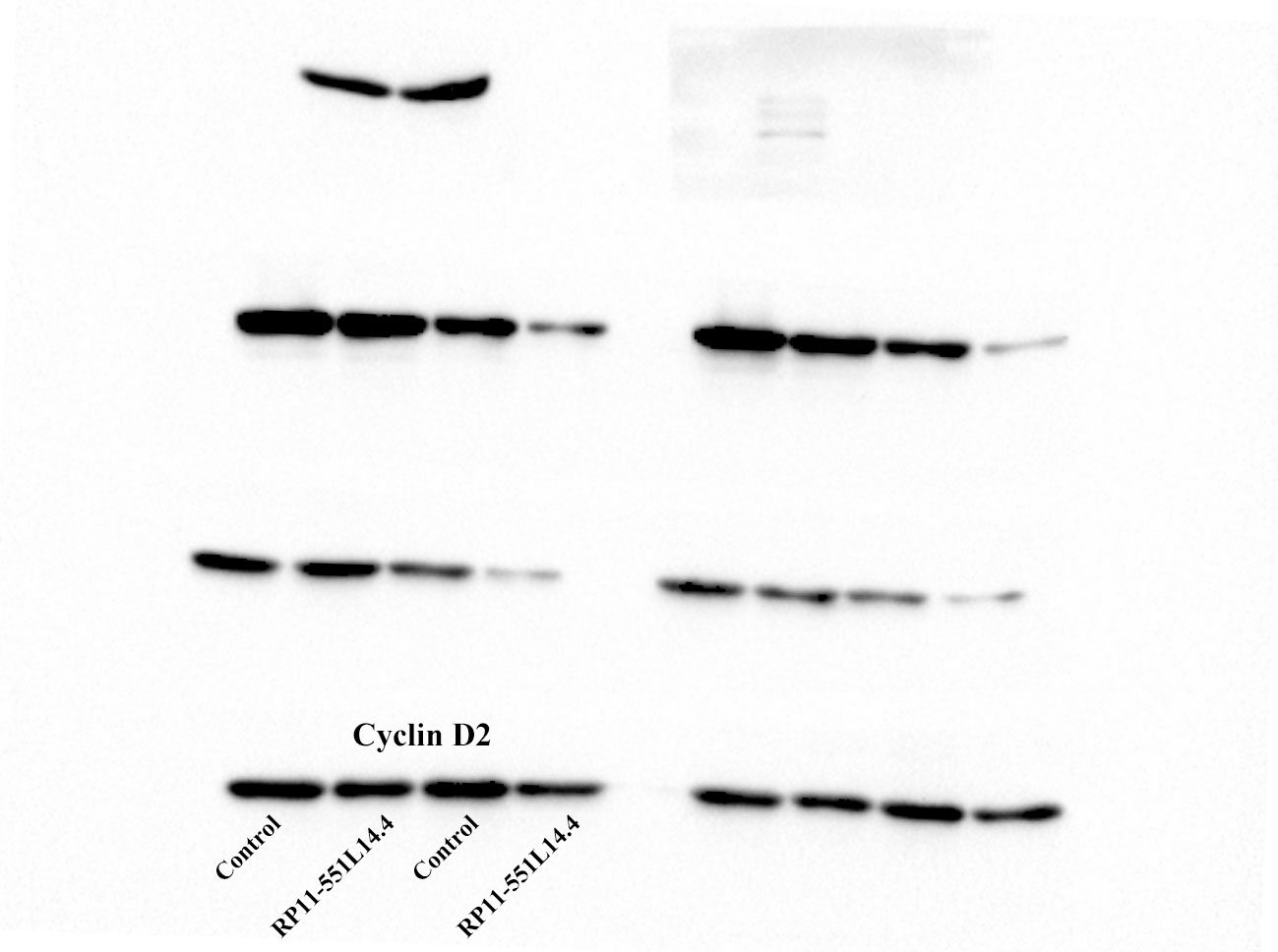


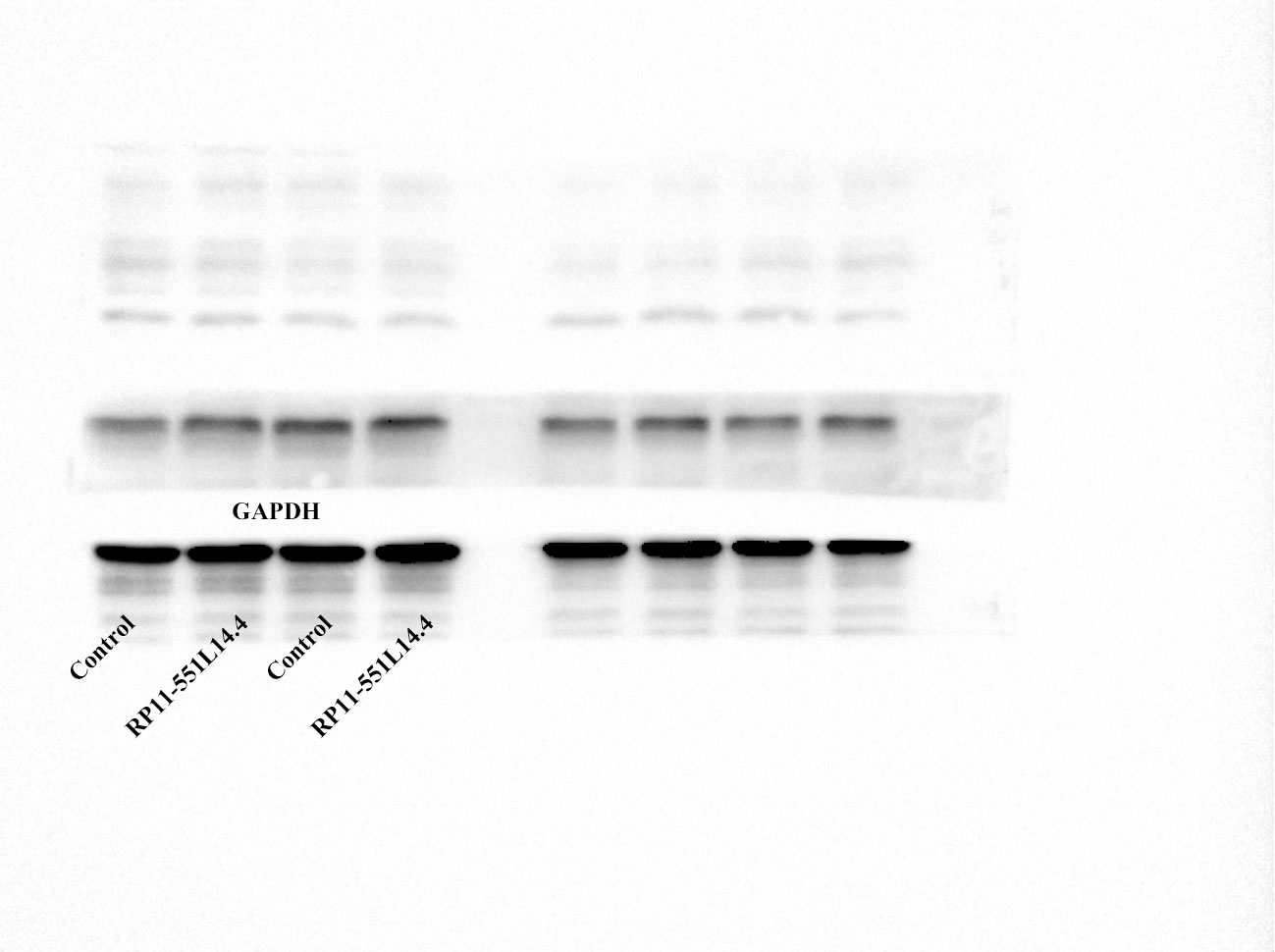

Supplement: Supplemental Information 1 [file peerj-10-14482-s001.docx]
